# Special Issue “Feature Papers in Biosensors Section 2022”

**DOI:** 10.3390/s23073704

**Published:** 2023-04-03

**Authors:** Huangxian Ju, Nicole Jaffrezic-Renault

**Affiliations:** 1 State Key Laboratory of Analytical Chemistry for Life Science, School of Chemistry and Chemical Engineering, Nanjing University, Nanjing 210023, China; 2 Institute of Analytical Sciences, University of Lyon, UMR CNRS 5280, 5 Rue de La Doua, 69100 Villeurbanne, France

Biosensors are devices composed of a biorecognition part and of a transduction part. Natural biomolecules such as enzymes [1], antibodies [2], DNA strains [3], bacteria and yeast [4,5], cells and even plant/animal tissues can constitute the recognition part. Synthesized polymers such as MIP (molecularly imprinted polymers) [1], aptamers and peptides can also be used as recognition part. In this special issue, molecularly imprinted overoxidized polypyrrole doped with copper nanoparticles was used for the electrochemical detection of sulfadiazine [6]. When natural biomolecules are used, one of the key points is the maintenance of the activity of the biomolecule after their immobilization on the transducer surface. Achitosan matrix was demonstrated to maintain enzyme activity for a longer period of time [1]. The stability of non-covalent avidin-biotin binding is widely utilized for anchoring biomolecules, and neutravidin and streptavidin are two commonly used avidin analogues. It was demonstrated that streptavidin is preferable to neutravidin for constructing lipid bilayers based sensing platforms [7]. Nanostructures based on framework DNA hold excellent promise for molecular biology studies and versatile tools for biosensor applications as reviewed in [3]. Dynamic mode decomposition of fluorescence loss was also used for monitoring protein diffusion, protein assemblies and protein aggregates in living cells [8]. Biosensors based on immobilized bacteria or yeast can be used for toxicological monitoring in natural waters [4] or for determining biochemical oxygen demand [5]. 3D-printing leads to low-cost biosensors, such as an immunosensor for the rapid detection of *Escherichia coli* bacteria [2]. A multiplexed bioluminescent assay based on 3D microtissues was performed for monitoring inflammatory, antioxidant bioactivities, presence of heavy metals and toxicity [9]. After an overview of the important biomarkers for several common children cancers, the developed biosensors for early detection of pediatric cancer are enumerated [10]. A review is conducted on the progress of the use of low-cost sensors in healthcare monitoring, the algorithms used to process sensor data and the wireless communication techniques [11].

Non-contact and non-invasive techniques are used for monitoring some behaviors or some health problems. They are also classified in biosensing techniques. A non-invasive measurement of the arterial blood based on a diffused light model has been presented [12]. Infra-red thermal imaging with heart rate variability was used to obtain features related to the psychophysiology of drivers, and data were analyzed by machine learning analyzers [13,14]. Wearable inertial measurement units associated with machine learning algorithm were used to predict Minimum Foot Clearance (MFC) timing [15]. These non-invasive techniques can also be used for monitoring the quality of agrifood products. The digital assessment and classification of wine fault were obtained using a low-cost electronic nose based on near-infrared spectroscopy and machine learning modelling [16].

## References

[1] Chmayssem A., Shalayel I., Marinesco S., Zebda A. (2023). Investigation of GOx Stability in a Chitosan Matrix: Applications for Enzymatic Electrodes. Sensors.

[2] Malhotra S., Pham D.S., Lau M.P.H., Nguyen A.H., Cao H. (2022). A Low-Cost, 3D-Printed Biosensor for Rapid Detection of Escherichia coli. Sensors.

[3] Liu B., Wang F., Chao J. (2023). Programmable Nanostructures Based on Framework-DNA for Applications in Biosensing. Sensors.

[4] Kharkova A., Arlyapov V., Medvedeva A., Lepikash R., Melnikov P., Reshetilov A. (2022). Mediator Microbial Biosensor Analyzers for Rapid Determination of Surface Water Toxicity. Sensors.

[5] Kurbanalieva S., Arlyapov V., Kharkova A., Perchikov R., Kamanina O., Melnikov P., Popova N., Machulin A., Tarasov S., Saverina E. (2022). Electroactive Biofilms of Activated Sludge Microorganisms on a Nanostructured Surface as the Basis for a Highly Sensitive Biochemical Oxygen Demand Biosensor. Sensors.

[6] Elamin M.B., Ali S.M.A., Essousi H., Chrouda A., Alhaidari L.M., Jaffrezic-Renault N., Barhoumi H. (2023). An Electrochemical Sensor for Sulfadiazine Determination Based on a Copper Nanoparticles/Molecularly Imprinted Overoxidized Polypyrrole Composite. Sensors.

[7] Sut T.N., Park H., Koo D.J., Yoon B.K., Jackman J.A. (2022). Distinct Binding Properties of Neutravidin and Streptavidin Proteins to Biotinylated Supported Lipid Bilayers: Implications for Sensor Functionalization. Sensors.

[8] Wüstner D. (2022). Dynamic Mode Decomposition of Fluorescence Loss in Photobleaching Microscopy Data for Model-Free Analysis of Protein Transport and Aggregation in Living Cells. Sensors.

[9] Calabretta M.M., Gregucci D., Guarnieri T., Bonini M., Neri E., Zangheri M., Michelini E. (2022). Bioluminescence Sensing in 3D Spherical Microtissues for Multiple Bioactivity Analysis of Environmental Samples. Sensors.

[10] Gharehzadehshirazi A., Zarejousheghani M., Falahi S., Joseph Y., Rahimi P. (2023). Biomarkers and Corresponding Biosensors for Childhood Cancer Diagnostics. Sensors.

[11] Liu Z., Cascioli V., McCarthy P.W. (2023). Healthcare Monitoring Using Low-Cost Sensors to Supplement and Replace Human Sensation: Does It Have Potential to Increase Independent Living and Prevent Disease?. Sensors.

[12] Zhang A.C., Lo Y.-H. (2022). Non-Invasive Blood Flow Speed Measurement Using Optics. Sensors.

[13] Cardone D., Perpetuini D., Filippini C., Mancini L., Nocco S., Tritto M., Rinella S., Giacobbe A., Fallica G., Ricci F. (2022). Classification of Drivers’ Mental Workload Levels: Comparison of Machine Learning Methods Based on ECG and Infrared Thermal Signals. Sensors.

[14] Filippini C., Di Crosta A., Palumbo R., Perpetuini D., Cardone D., Ceccato I., Di Domenico A., Merla A. (2022). Automated Affective Computing Based on Bio-Signals Analysis and Deep Learning Approach. Sensors.

[15] Asogwa C.O., Nagano H., Wang K., Begg R. (2022). Using Deep Learning to Predict Minimum Foot–Ground Clearance Event from Toe-Off Kinematics. Sensors.

[16] Gonzalez Viejo C., Fuentes S. (2022). Digital Assessment and Classification of Wine Faults Using a Low-Cost Electronic Nose, Near-Infrared Spectroscopy and Machine Learning Modelling. Sensors.

